# The Separation of Sulfide Minerals from Fluorapatite Ore in Acidic De-Magnesium Flotation Process

**DOI:** 10.3390/ma19081633

**Published:** 2026-04-18

**Authors:** Long Luo, Mianyan Yang, Hong Zhang, Lang Yang, Feng Rao

**Affiliations:** 1 Zijin School of Geology and Mining, Fuzhou University, Fuzhou 350108, China; 231620037@fzu.edu.cn (L.L.); siryanglang@fzu.edu.cn (L.Y.); 2 State Key Laboratory of Green and Efficient Development of Phosphorus Resources, Guiyang 550014, China; zhanghong_pz3a@chinagpc.com; 3 Fujian Provincial Key Laboratory of Green Extraction and High-Value Utilization of New Energy Metals, Fuzhou 350108, China; 4 Guizhou Phosphate Chemical Group Co., Ltd., Guiyang 550001, China

**Keywords:** phosphate flotation, sulfide minerals, surface chemistry, adsorption

## Abstract

In this study, the characteristics of sulfide minerals during the acidic double reverse flotation of phosphate ore and the adsorption mechanisms of sodium oleate (NaOL) and dodecyl trimethyl ammonium bromide (DTAB) were investigated. Micro-flotation test results indicated that NaOL effectively collected galena, sphalerite, and pyrite at a concentration of 1 × 10^−3^ mol/L and pH 4–5.5, whereas DTAB exhibited selectivity for galena at 1 × 10^−4^ mol/L. Mixed mineral flotation revealed that NaOL induced a non-selective bulk flotation of sulfides with dolomite, resulting in a high froth yield of 93.23%, while the DTAB system showed superior selectivity with a froth yield of 54.91%. Surface analyses (Zeta potential, FTIR, and XPS) confirmed that NaOL chemisorbs onto sulfide surfaces via metal-oleate complexes, whereas DTAB adsorption is dominated by electrostatic attraction. Bench-scale tests validated the “double-rejection” flowsheet, significantly upgrading the P_2_O_5_ grade from 23.38% to 31.47% by sequentially partitioning Pb, Zn and Fe into the froth tailings. Size-by-assay analysis indicated that the sulfide separation was primarily controlled by the extent of mineral liberation. These findings provide a robust theoretical framework and practical guidance for the simultaneous management of sulfide minerals during phosphate beneficiation.

## 1. Introduction

Selective separation of collophane from calcareous (predominantly dolomite) and siliceous gangue is essential for producing phosphate concentrates suitable for wet-process phosphoric acid (WPA) and fertilizer manufacturing [1,2]. Among established routes, the acidic double-reverse flotation flowsheet has become a dominant industrial practice, particularly for refractory ores [3,4]. This flowsheet comprises an initial anionic reverse flotation stage (pH 4.0–5.5) using fatty acids to remove dolomite while fluorapatite is depressed by inorganic acids (e.g., H_2_SO_4_ or H_3_PO_4_) [5,6]. This is followed by a cationic reverse flotation stage employing amines to remove silicates via electrostatic adsorption [7,8]. The viability of this flowsheet is well established. In the beneficiation of a refractory ore from Guizhou, China, the P_2_O_5_ grade was enriched from 22.45% to 30.68%, alongside a significant reduction in MgO and SiO_2_ contents [9].

Despite the proven efficacy of this route in de-magnesium and desilication, the behavior of associated sulfide minerals (e.g., pyrite, sphalerite, galena) remains largely unexplored. Sulfides exert detrimental effects that are disproportionate to their mass fraction. Primarily, they serve as the main carriers of hazardous heavy metals, such as lead (Pb), zinc (Zn) and cadmium (Cd), which pose severe challenges in meeting the increasingly stringent regulatory limits for phosphate fertilizers [10,11]. Furthermore, during the acidulation stage of WPA production, sulfide dissolution creates a reducing environment. This not only accelerates the pitting corrosion of process equipment but also adversely modifies the crystallization habit and filtration kinetics of phosphogypsum [12,13]. Current gangue rejection methodologies often rely on pre-treatment. While thermal calcination effectively decomposes sulfides, its industrial viability is constrained by high energy intensity and flue gas treatment costs [14]. The conventional approach employs a pre-flotation stage using xanthate collectors to selectively remove sulfides prior to phosphate beneficiation [15,16]. This inevitably increases flowsheet complexity and operational costs. The characteristics of these sulfide minerals during the acidic double-reverse flotation process have yet to be fully investigated. It remains unclear whether interactions with the primary collectors (fatty acids and amines) induce the flotation or depression of these sulfides. Understanding these underlying mechanisms is essential to determine whether sulfides can be rejected simultaneously with dolomite or siliceous gangue.

The present study systematically investigates the deportment and surface chemistry of sulfide minerals within a fluorapatite ore processed by an acidic double-reverse flotation flowsheet. A multi-scale approach was adopted to elucidate the separation mechanisms. Micro-flotation tests combined with surface analyses (Zeta potential, FTIR, and XPS) were employed. NaOL and DTAB were used as model collectors representing typical fatty acids and amines, to decouple complex interfacial interactions and compare adsorption on sulfide surfaces. Artificial mixed mineral experiments were performed to evaluate the flotation selectivity and competitive separation behavior of sulfides under different collector regimes. These fundamental insights were validated in an actual ore system, where the partitioning and mineralogical associations of Pb and Zn were quantified using inductively coupled plasma (ICP) analysis and SEM-EDS. This study aims to establish a mechanistic framework for a novel “double-rejection” mechanism, offering practical guidance for the simultaneous control of associated sulfide minerals during phosphate beneficiation.

## 2. Materials and Methods

### 2.1. Materials

A representative actual collophanite ore sample was sourced from the Wengfu phosphate deposit in Guizhou Province, China. For micro-flotation and mechanistic studies, high-purity mineral specimens were obtained from various sources in China: pyrite from Hubei Province, galena and sphalerite from Hunan Province, dolomite and quartz from Yunnan Province. Each pure mineral sample was individually comminuted using a mechanical agate mortar (Fritsch, Idar-Oberstein, Germany) and subsequently dry-sieved to isolate the −74 + 38 μm size fraction.

NaOL and DTAB of ACS reagent grade were purchased from Aladdin Industrial Corporation (Shanghai, China), and utilized as collectors. Distilled water was used throughout all flotation experiments. The pulp pH was adjusted using analytical grade dilute H_2_SO_4_ and NaOH solutions. Sulfuric acid (H_2_SO_4_, analytical grade, 98%) and sodium hydroxide (NaOH, analytical grade, ≥99%) were purchased from Aladdin Industrial Corporation, China. Hydrochloric acid (HCl, analytical grade, 37%) and potassium chloride (KCl, analytical grade, ≥99.5%) used for zeta potential measurements were purchased from Aladdin Industrial Corporation, China.

### 2.2. Methods

**Figure 1:**
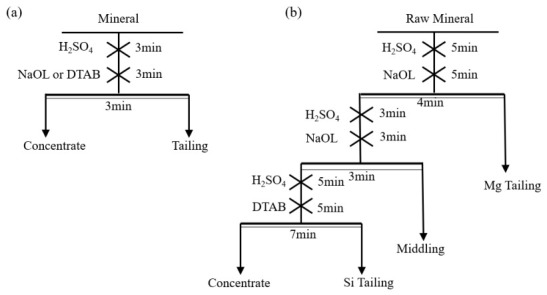
Schematic Flowsheets of (**a**) the micro-flotation test and (**b**) the bench-scale double reverse flotation.

Micro-flotation experiments were conducted employing an XFG-II mechanical (Jilin Exploration Machinery Plant, Changchun, China) agitation flotation apparatus featuring an 80 mL chamber volume, operated at an impeller speed set to 1992 rpm. For each test, 4 g of the mineral sample was dispersed in deionized water and conditioned to ensure a uniform suspension. The pulp pH was regulated using dilute H_2_SO_4_ or NaOH solutions and equilibrated for 3 min. Subsequently, the collector, either NaOL or DTAB, was introduced and conditioned for an additional 3 min. The corresponding flowsheet is illustrated in Figure 1a. For artificial mixed mineral flotation, the procedure was identical to that of the single mineral tests, except that the mineral components were blended in specific mass ratios. Mixed Ore I comprised apatite, dolomite, pyrite, galena, and sphalerite at a mass ratio of 5:3:1:1:1, whereas Mixed Ore II consisted of apatite, quartz, pyrite, galena, and sphalerite at a ratio of 5:2:1:1:1. Following a 3 min froth collection period, both the floated and non-floated fractions were separately filtered, dried, and weighed. Flotation recovery and mass yield were calculated based on the mass balance between the two products.

Bench-scale experiments were conducted using an XFD laboratory flotation machine (XFD-IV, Jilin Exploration Machinery Plant, Changchun, China) equipped with a 1.5 L cell, operating at 1992 rpm with a pulp density of 30 wt.%. The double reverse flotation flowsheet was initiated with an anionic stage for dolomite rejection at pH 5 (adjusted with H_2_SO_4_). During this stage, NaOL was introduced step-wise. The cell underflows from multiple batches were then bulked and homogenized to serve as the feed for the subsequent cationic desilication stage, which utilized DTAB for siliceous gangue rejection. This bench-scale flowsheet is depicted in Figure 1b. Both the froth tailings and the final concentrates were filtered, dried, and assayed to evaluate overall metallurgical performance.

The mineralogical phases and purity of the samples were analyzed using an X-ray diffractometer (Bruker D8 Advance, Karlsruhe, Germany) equipped with Cu Kα radiation (λ = 1.5406 Å). The operating voltage and current were set to 40 kV and 40 mA, respectively. The measurements were conducted over a 2θ range of 5° to 70° with a scanning rate of 2°/min. To determine the phase purity and identify the specific minerals present (i.e., pyrite, galena, sphalerite, quartz, and dolomite), the obtained experimental diffraction patterns were compared against standard reference patterns from the Joint Committee on Powder Diffraction Standards (JCPDS) database.

The electrokinetic properties of the sulfide minerals (galena, sphalerite, and pyrite) were measured with a Malvern Zetasizer Nano ZS90 analyzer (Malvern Instruments, Malvern, UK). Mineral suspensions were prepared by dispersing 50 mg of fine particles (−5 μm) in 50 mL of background electrolyte solution (1 × 10^−3^ mol/L KCl). The pH was adjusted using HCl or NaOH solutions. For measurements involving collectors, the minerals were conditioned with NaOL (1 × 10^−3^ mol/L) or DTAB (1 × 10^−4^ mol/L) for 3 min prior to analysis. The average zeta potential was recorded from three independent measurements to ensure data reproducibility.

The interactions between the mineral surfaces and collectors were characterized via FTIR spectroscopy (Nicolet Avatar 370, Thermo, CA, USA) and XPS (ESCALAB 250Xi, Thermo Fisher Scientific, Waltham, MA, USA) FTIR transmittance spectra were recorded over a wavenumber range of 4000–500 cm^−1^ with a resolution of 2 cm^−1^. For XPS measurements, 2 g of the specific −5 μm sulfide mineral was dispersed in 25 mL of deionized water. The suspension pH was adjusted to 5, followed by the addition of NaOL (1 × 10^−3^ mol/L) for the treated samples. The pulp was conditioned for 3 min to reach adsorption equilibrium. Subsequently, the solids were separated by filtration, rinsed three times with deionized water to remove weakly entrained species, and vacuum-dried at room temperature (25 °C) to prevent surface oxidation. XPS spectra were acquired using a Thermo Fisher ESCALAB 250Xi spectrometer equipped with a monochromatic Al Kα X-ray excitation source (1486.6 eV). The instrument was operated at 150 W (15 kV, 10 mA) with a constant analyzer pass energy. All binding energies were calibrated by referencing the adventitious carbon (C1s) peak at 284.8 eV to compensate for surface charging effects. Data acquisition, spectral deconvolution and quantification were performed using the Avantage software (version 5.9904, Thermo Fisher Scientific, Waltham, MA, USA) package.

The particle size fraction used for SEM-EDS analysis (Vega, TESCAN, Brno, Czech Republic) was consistent with that of the bench-scale flotation tests. Samples were mounted on a sample stub using conductive carbon tape, and excess non-adhering powder was carefully removed. Following gold sputtering to minimize charging effects, the samples were analyzed using a Vega SEM operating at 15 kV. EDS spectra were collected under the same beam conditions used for imaging, with a live-time of 40 s.

## 3. Results and Discussion

### 3.1. Characterization of Raw Materials

**Figure 2:**
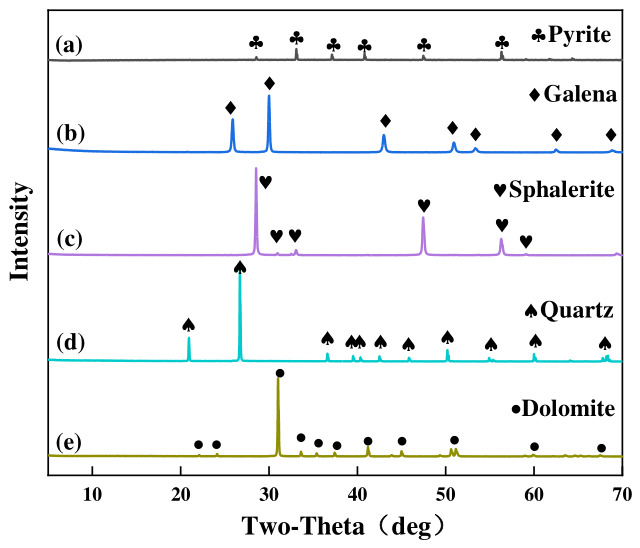
XRD patterns of the pyrite (a), galena (b), sphalerite (c), quartz (d), and dolomite (e) samples.

The crystallographic composition and phase purity of these size fractions were rigorously validated by X-ray diffraction (XRD) analysis, confirming that all pure mineral samples possessed a purity exceeding 95% (Figure 2).

**Figure 3:**
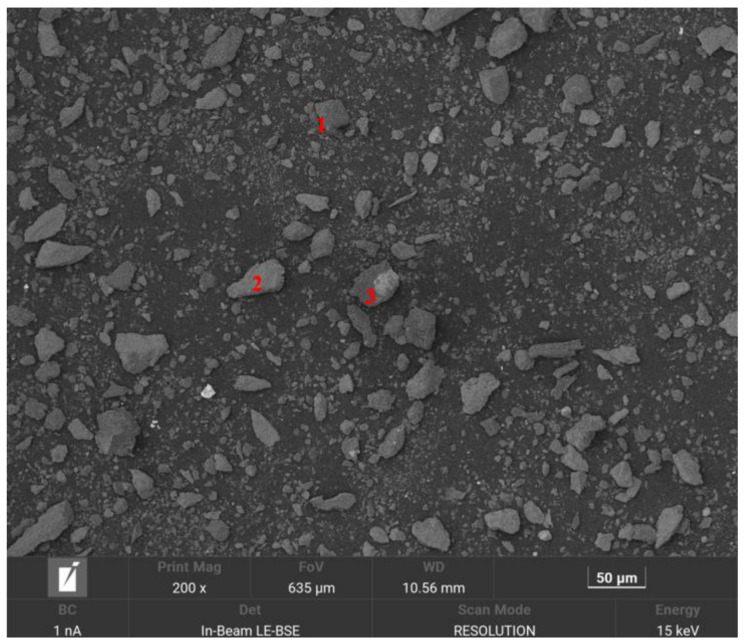
SEM micrograph of the run-of-mine ore. The red numbers (1, 2, and 3) indicate the specific locations of the EDS point analyses corresponding to the data in Table 1.

As illustrated in Figure 3, the SEM-EDS characterization of the run-of-mine ore revealed an elemental assemblage of Pb, Zn, Fe, and S. Specifically, the red numbers 1, 2, and 3 in the micrograph denote distinct point-scanning locations where EDS analysis was conducted on the raw ore surface. The corresponding elemental compositions at these points (detailed in Table 1) verify the presence of lead, zinc, iron, and sulfur. This strong elemental association indicates that these metals exist primarily as associated sulfide minerals (e.g., galena, sphalerite, and pyrite). Consequently, within the mineral matrix, they are present in their respective ionic forms.

### 3.2. Micro-Flotation Tests

**Figure 4:**
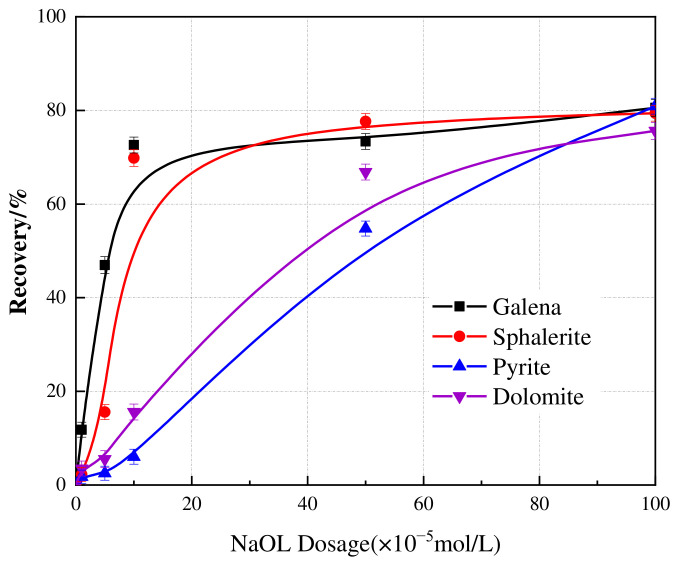
Effect of NaOL dosage on the flotation recovery of galena, sphalerite, pyrite, and dolomite. The scatter points represent the experimental data, and the solid lines are drawn to guide the eye. Error bars indicate the standard deviation of independent experiments.

To elucidate the deportment of associated sulfide minerals during this stage, their flotation behavior was systematically evaluated using NaOL as a representative collector. Figure 4 shows the flotation recovery of galena, sphalerite, pyrite and dolomite as a function of NaOL dosage at pH 5. The recoveries of all four minerals presented a positive correlation with NaOL dosage, reflecting the strong collecting power of this fatty acid. Galena and sphalerite exhibited high floatability, reaching recoveries of 72% and 70% at a dosage of 1 × 10^−4^ mol/L. Dolomite and pyrite exhibited a more gradual linear increase. At a high dosage of 1 × 10^−3^ mol/L, the recoveries of all minerals were reached 75–80%.

**Figure 5:**
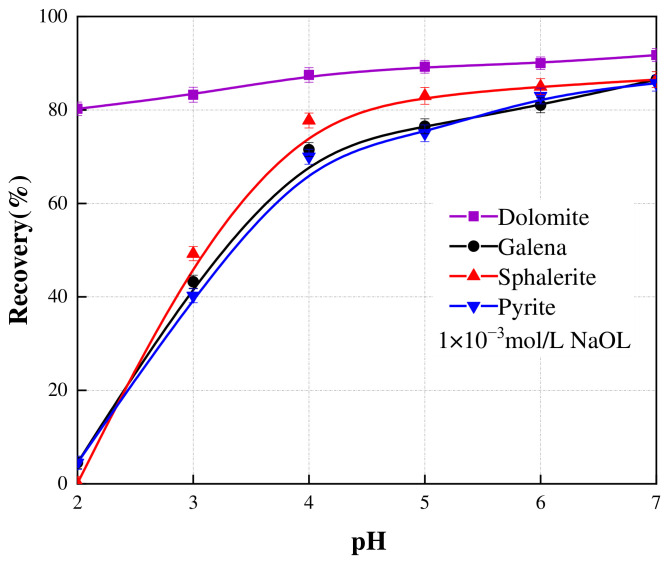
Effect of various pH on the flotation recovery of galena, sphalerite, pyrite, and dolomite. The scatter points represent the experimental data, and the solid lines are drawn to guide the eye. Error bars indicate the standard deviation of independent experiments.

The influence of pH on the floatability of these minerals in the 1 × 10^−3^ mol/L NaOL system is shown in Figure 5. Dolomite maintained excellent floatability (>80% recovery) across the entire tested pH range (2–7). In contrast, the responses of the sulfide minerals were highly pH-dependent. Under acidic conditions (pH 2), the sulfide minerals were effectively depressed, yielding recoveries below 5%. Within the standard operational pH range of the anionic reverse flotation flowsheet (pH 4.0–5.5), the recoveries of galena, sphalerite, and pyrite increased substantially, approaching the recovery of dolomite. These findings underscore the poor selectivity of NaOL between dolomite and the associated sulfide minerals. While typically detrimental in conventional single-mineral recovery systems, this non-selective co-collection behavior proves strategically advantageous for the proposed double-reverse flowsheet, as it enables the simultaneous bulk rejection of these impurities into the initial tailings. The underlying mechanism governing this pronounced co-flotation is the strong chemisorption of oleate species onto the metallic active sites, a phenomenon comprehensively corroborated by subsequent surface analyses.

**Figure 6:**
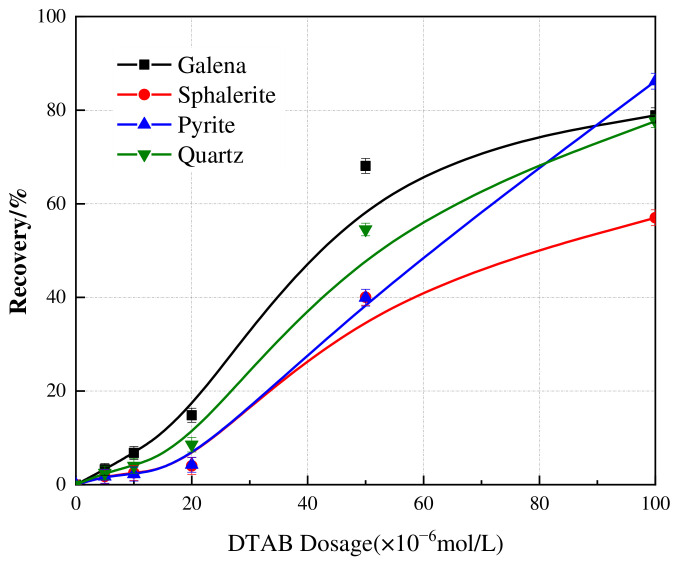
Effect of DTAB dosage on the flotation recovery of galena, sphalerite, pyrite, and quartz. The scatter points represent the experimental data, and the solid lines are drawn to guide the eye. Error bars indicate the standard deviation of independent experiments.

The subsequent stage of the flowsheet utilizes cationic collectors to remove siliceous gangue. Figure 6 presents the flotation recoveries of the sulfides and quartz as a function of DTAB dosage at pH 5. Compared to NaOL, the DTAB system showed distinct selectivity. Galena exhibited superior floatability at lower dosages, with recovery rising sharply to 68% recovery at 5 × 10^−5^ mol/L, surpassing all other minerals. Pyrite displayed a delayed response, remaining strongly depressed (<5%) at dosages below 2 × 10^−5^ mol/L. However, its recovery escalated sharply at higher concentrations, eventually peaking at 86%. Conversely, sphalerite showed the weakest flotation response, with recovery peaking at 57%. Quartz presented a steady increase in recovery of 77%.

**Figure 7:**
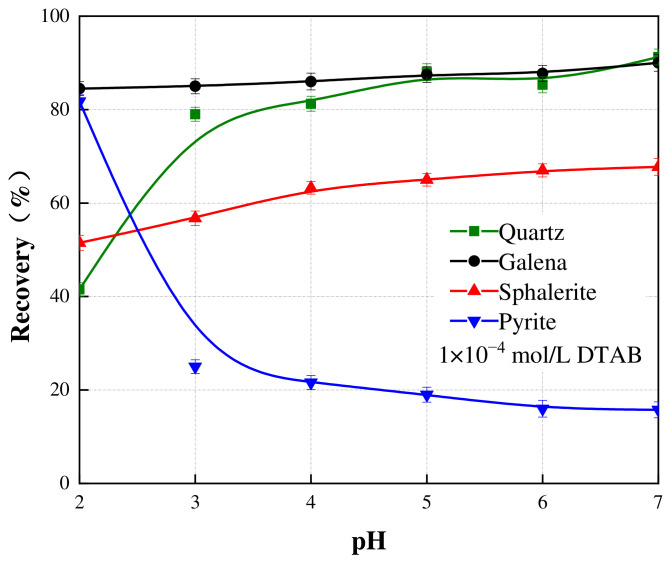
Effect of various pH on the flotation recovery of galena, sphalerite, pyrite, and quartz. The scatter points represent the experimental data, and the solid lines are drawn to guide the eye. Error bars indicate the standard deviation of independent experiments.

The pH-dependent flotation behavior in the DTAB system (1 × 10^−4^ mol/L) highlighted distinct responses among the minerals (Figure 7). Galena showed a robust flotation performance, maintaining a high recovery (>85%) across the entire pH range. Quartz recovery increased from 42% at pH 2 to a maximum of 90% at pH 7, confirming the suitability of DTAB for desilication. Pyrite showed a completely opposite trend. Its recovery decreased from 82% at pH 2 to below 20% at pH 7. Sphalerite showed a moderate increase from 51% to 68% across the same pH range.

### 3.3. Mixed Minerals Flotation Tests

The experimental data for the artificial mixed mineral flotation are presented in Table 2. Mixture I was composed of apatite, dolomite, and sulfides at a ratio of 5:3:3. Upon the addition of 1 × 10^−3^ mol/L NaOL at pH 5, the system exhibited a remarkably high froth yield of 93.23%. Given that dolomite and sulfides constitute only 54% of the feed mass, this excessive yield indicates that NaOL acts as a non-selective collector, floating nearly all minerals including the valuable fluorapatite. Critically, for the purpose of impurity removal, this result confirms that sulfide minerals possess floatability comparable to dolomite.

Mixture II was composed of apatite, quartz, and sulfides at a ratio of 5:2:3. Theoretically, if perfect separation occurred where quartz and sulfides are floated while apatite is depressed, the expected mass yield of the froth product would be 50%. The experimental froth yield obtained with 1 × 10^−4^ mol/L DTAB was 54.91% (Table 2). This value aligns closely with the theoretical mass balance of the gangue and sulfides components. Unlike the bulk flotation observed with NaOL, the DTAB system presented excellent selectivity. It effectively collected the quartz and sulfide minerals while allowing the fluorapatite to remain in the concentrate.

The distinct mass distributions in these two systems provide a clear deportment for sulfide minerals. The high yield in the NaOL system indicates that a significant portion of sulfides is likely rejected in the first stage. Subsequently, the selective separation observed in the DTAB system suggests that any residual sulfides carried over to the second stage are effectively floated alongside the siliceous gangue. The distinct mass distributions in these two systems elucidate a clear deportment pathway for the associated sulfide minerals. Unlike conventional flowsheets that necessitate dedicated desulfurization stages employing toxic depressants, our findings demonstrate that sulfides can be effectively partitioned through a synergistic ‘double-rejection’ mechanism. Specifically, a substantial fraction of the sulfides is co-rejected with dolomite via NaOL-induced chemisorption, while the residual sulfides are subsequently scavenged alongside siliceous gangue through electrostatic interactions in the DTAB stage.

### 3.4. Zeta Potential Measurements

**Figure 8:**
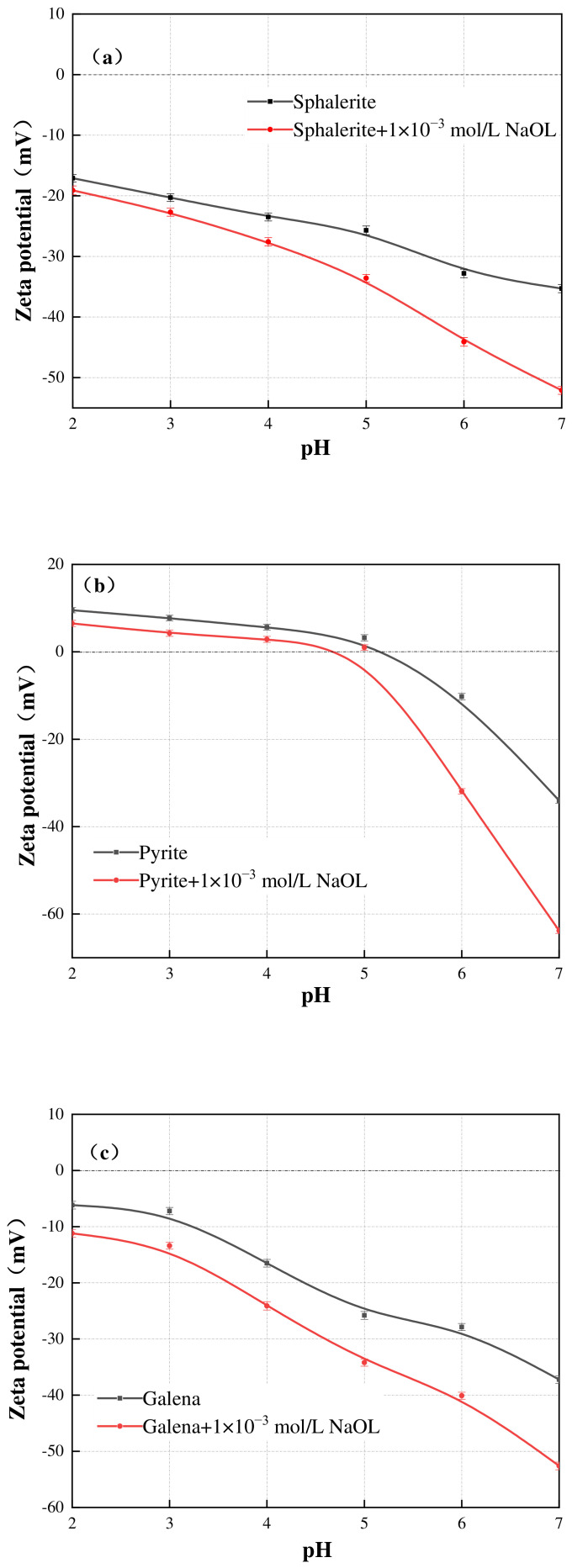
Zeta potential of sphalerite (**a**), pyrite (**b**) and galena (**c**) at various pH in the NaOL system. The scatter points represent the experimental data, and the solid lines are provided as visual guides. Error bars indicate the standard deviation of independent measurements.

Figure 8 shows the zeta potentials of sphalerite, pyrite, and galena as a function of pH (2–7) before and after conditioning with NaOL. In the NaOL system, the surfaces of sphalerite and galena presented consistently negative zeta potentials across the entire tested pH range, reaching approximately −25.7 mV and −25.8 mV at pH 5, respectively. Pyrite showed an isoelectric point (IEP) at roughly pH 5.2, aligning well with previous literature [17,18,19]. Upon the addition of 1 × 10^−3^ mol/L NaOL, the zeta potentials of all three sulfide minerals become more negative. Given that both the mineral surfaces and the oleate species are negatively charged, this pronounced negative shift indicates that the adsorption successfully overcame electrostatic repulsion. This behavior is a hallmark of strong specific adsorption (chemisorption) between the anionic oleate polar groups and the metallic active sites on the sulfide surfaces, thereby rendering the shear plane highly electronegative [20,21].

**Figure 9:**
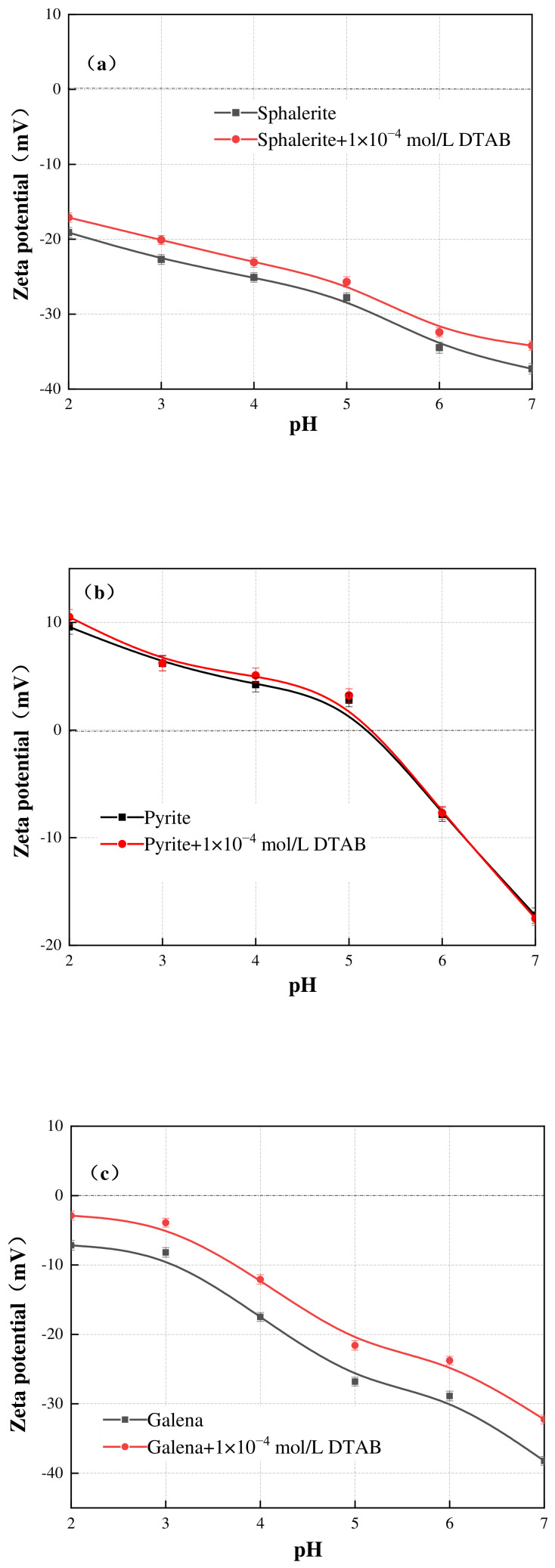
Zeta potential of sphalerite (**a**), pyrite (**b**) and galena (**c**) at various pH in the DTAB system. The scatter points represent the experimental data, and the solid lines are provided as visual guides. Error bars indicate the standard deviation of independent measurements.

Conversely, the electrokinetic behavior in the presence of DTAB reflects a fundamentally different interaction mechanism. Figure 9 shows the zeta potentials of the sulfides before and after conditioning with 1 × 10^−4^ mol/L DTAB. Following DTAB introduction, the zeta potentials of all three minerals become more positive. For instance, the zeta potential of galena increased from −17.5 mV to −12.1 mV at pH 4. Similarly, the inherently negative potentials of sphalerite and pyrite were increased. This is attributed to the charge neutralization effect caused by the adsorption of cationic surfactant heads (DTA^+^) onto the negatively charged mineral-water interfaces. Unlike the massive potential inversion observed with NaOL, the moderate shifts induced by DTAB strongly indicate that its interaction with the sulfide minerals is primarily influenced by electrostatic attraction (physisorption) rather than robust chemisorption [22,23].

### 3.5. FTIR Measurements

**Figure 10:**
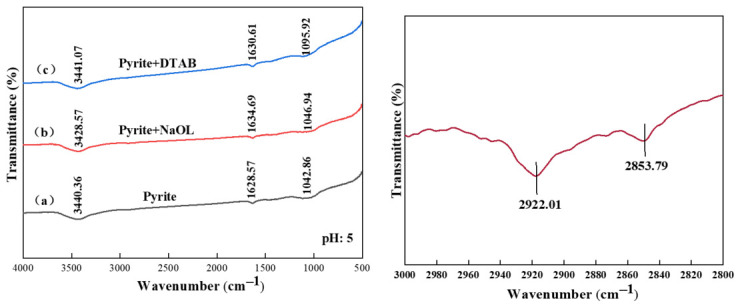
FTIR spectra of pyrite surface: (a) pristine, (b) treated with NaOL, (c) treated with DTAB and the enlarged view of the 3000–2800 cm^−1^ region for sodium oleate and pyrite.

**Figure 11:**
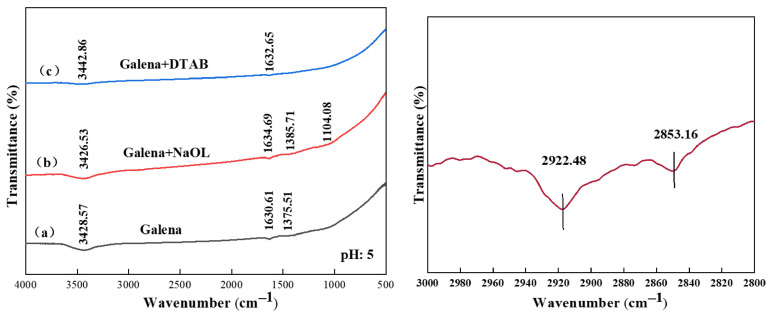
FTIR spectra of galena surface: (a) pristine, (b) treated with NaOL, and (c) treated with DTAB and the enlarged view of the 3000–2800 cm^−1^ region for sodium oleate and galena.

**Figure 12:**
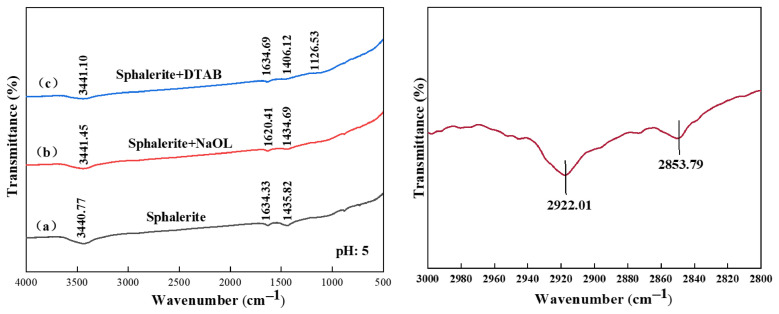
FTIR spectra of sphalerite surface: (a) pristine, (b) treated with NaOL, and (c) treated with DTAB and the enlarged view of the 3000–2800 cm^−1^ region for sodium oleate and sphalerite.

The FTIR spectra of the pure minerals exhibit characteristic diagnostic bands primarily associated with ambient surface hydration and inherent oxidation. As shown in Figure 10, Figure 11 and Figure 12. For pure sphalerite and galena, the broad band spanning 3400–3450 cm^−1^ and the distinctive peak at 1628–1634 cm^−1^ are assigned to the stretching and bending vibrations of adsorbed water molecules and surface hydroxyl (-OH) groups [24]. The spectrum of pure pyrite displays a prominent peak at 1042.86 cm^−1^, which is attributed to the asymmetric stretching vibration of surface sulfate species (SO_4_^2−^). This confirms the inevitable superficial oxidation of the sulfide surfaces upon exposure to atmospheric conditions during comminution and conditioning [25].

Upon conditioning with NaOL, significant spectral transformations occurred across the sulfide surfaces. New intense absorption bands emerged at approximately 2922 cm^−1^ and 2853 cm^−1^, corresponding to the asymmetric and symmetric stretching vibrations of the methylene (-CH_2_^−^) and methyl (-CH_3_^−^) groups of the oleate alkyl chain [26,27]. The appearance of these hydrophobic tails confirms the successful coverage of the collector on the mineral surfaces. Furthermore, taking sphalerite as a representative example, distinct new peaks manifested at 1620.41 cm^−1^ and 1434.69 cm^−1^. These bands are the classic signatures of the asymmetric and symmetric stretching vibrations of the carboxylate (-COO^−^) headgroup [28]. Crucially, the conspicuous absence of the carbonyl stretching band at 1710 cm^−1^ which characterizes free liquid oleic acid provides compelling evidence that NaOL did not merely precipitate on the surface. Instead, the oleate anions adsorbed via chemical coordination between the carboxylate headgroups and the metallic active sites (e.g., Zn^2+^ and Pb^2+^), forming highly hydrophobic metal-oleate surface complexes [20,29].

The interaction landscape was altered when DTAB was introduced. For instance, on the sphalerite surface, a newly formed band at 1406.12 cm^−1^, assigned to the C-N stretching vibration of the quaternary ammonium headgroup, was detected. For pyrite, the characteristic sulfate peak shifted from 1042.86 cm^−1^ to 1095.92 cm^−1^, accompanied by an increase in intensity. In the case of galena, although the hydrophobic alkyl chain bands were clearly visible, no new peaks associated with Pb-ligand covalent bonding were detected. The spectral features of the galena surface remained largely preserved, suggesting that DTAB adsorption is driven primarily by electrostatic attraction between the cationic headgroups and the negatively charged mineral surface. These spectral perturbations, without the formation of new strong covalent coordination bonds typical of chemisorption, indicate that the cationic collector (DTA^+^) primarily anchors to the negatively charged oxidation products (e.g., SO_4_^2−^) and localized electronegative domains via electrostatic attraction and hydrogen bonding networks [30,31,32,33].

### 3.6. XPS Analysis

**Figure 13:**
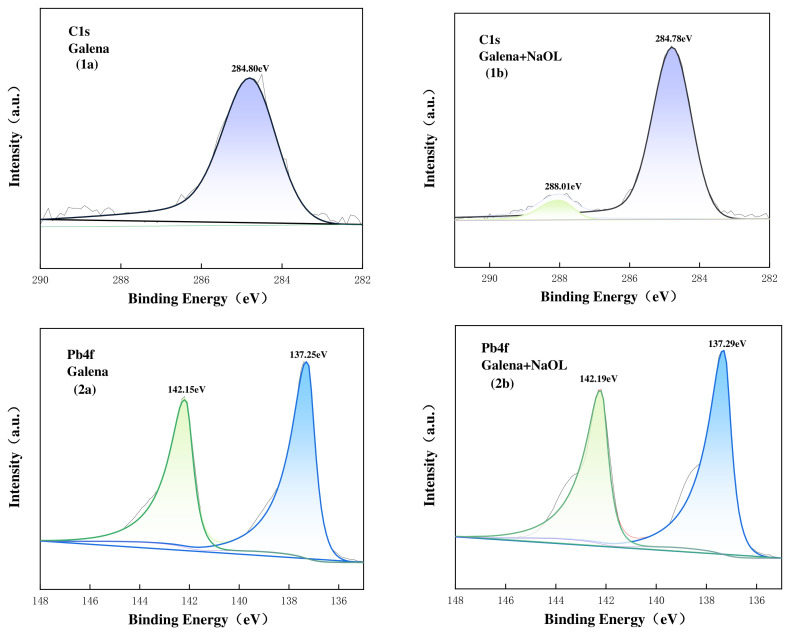
C1s, Pb4f XPS spectra on galena surface untreated (**1a**,**2a**), treated with NaOL (**1b**,**2b**). The different colored lines and shaded areas represent the deconvoluted component peaks.

**Figure 14:**
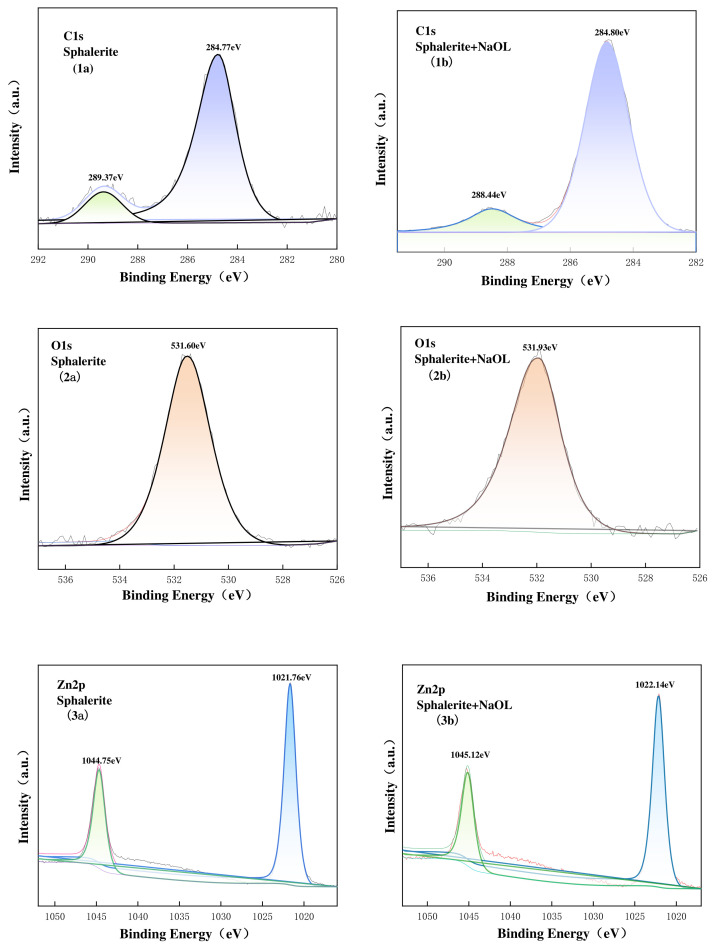
C1s, O1s and Zn2p XPS spectra on sphalerite surface untreated (**1a**,**2a**,**3a**), treated with NaOL (**1b**,**2b**,**3b**). The different colored lines and shaded areas represent the deconvoluted component peaks.

**Figure 15:**
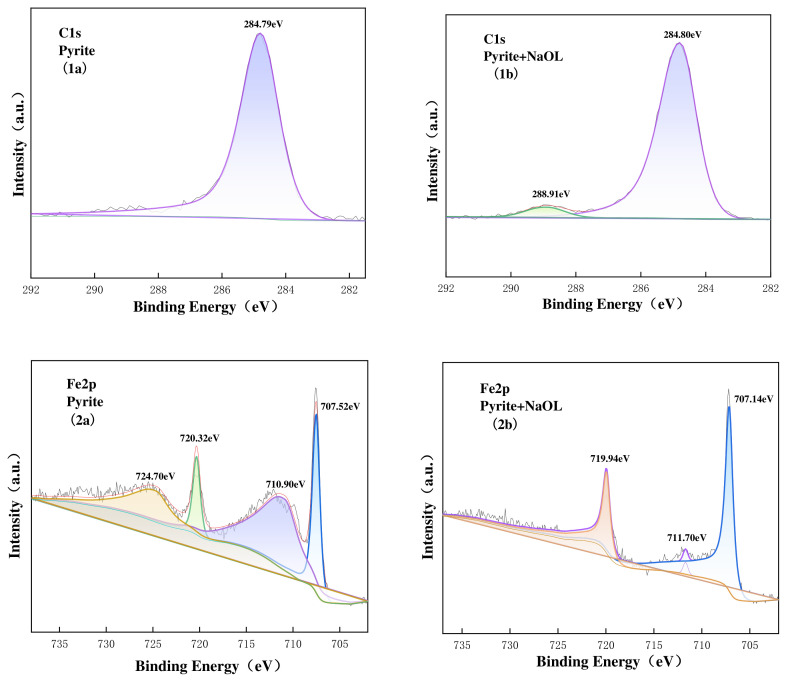
C1s, Fn2p XPS spectra on pyrite surface untreated (**1a**,**2a**), treated with NaOL (**1b**,**2b**). The different colored lines and shaded areas represent the deconvoluted component peaks.

As prior zeta potential and FTIR analyses indicated that DTAB adsorption was primarily governed by electrostatic physical interactions without altering the intrinsic chemical states of the surface atoms, the XPS investigation focused on the chemisorption behavior in the NaOL system. The high-resolution C1s spectra of pure and NaOL-treated galena, sphalerite, and pyrite surfaces are presented in Figure 13, Figure 14 and Figure 15. For all pristine minerals, the C1s spectra were dominated by a solitary peak centered at approximately 284.8 eV, which is universally attributed to the C-C and C-H bonds of adventitious carbon contamination [34]. Following NaOL treatment, a distinct new peak emerged at higher binding energies across all three minerals: 288.01 eV for galena, 288.44 eV for sphalerite, and 288.91 eV for pyrite. This new peak can be assigned to the carbon atoms in the carboxylate group (-COO^−^) of adsorbed oleate species. The appearance of this carboxylate-carbon signal provides direct evidence that the NaOL collector was adsorbed onto the surfaces of all three sulfide minerals, consistent with chemisorption involving surface metal sites and oleate species [35,36,37].

For galena (Figure 13), the high-resolution Pb 4f spectra of the pure mineral presented the characteristic doublet of Pb 4f_7/2_ and Pb 4f_5/2_ at 137.49 eV and 142.36 eV, respectively, corresponding to Pb^2+^ state on the pristine sulfide surface. Upon conditioning with NaOL, these peaks exhibited uniform shift to higher binding energies, shifting to 137.64 eV and 142.50 eV. This positive binding energy shift, similar to that observed for zinc in sphalerite, indicates a tangible decrease in the electron density of the surface Pb sites. This electron withdrawal is driven by the coordination with the highly electronegative carboxylate oxygens of the collector. Consequently, this provides robust evidence that the oleate anions coordinated with the Pb^2+^ sites to form a stable, hydrophobic lead-oleate surface complex.

For sphalerite (Figure 14), the O 1s peak shifted by 0.33 eV towards a higher binding energy (from 531.60 eV to 531.93 eV) after NaOL addition. Correspondingly, the Zn 2p peak, originally located at 1021.76 eV for pure sphalerite [38], shifted to a higher binding energy of 1022.14 eV. This positive shift (+0.38 eV) is consistent with a decrease in the electron density of the surface Zn sites caused by coordination between oxygen-containing ligand groups and surface Zn atoms on sphalerite [39]. This confirms the formation of robust, hydrophobic zinc-oleate surface complexes via chemisorption [28].

For pyrite (Figure 15), the Fe 2p spectra of the pure mineral exhibited the characteristic doublet of Fe 2p_3/2_ (707.52 eV) and Fe 2p_1/2_ (720.32 eV) assigned to pyrite [40]. After interaction with NaOL, the Fe 2p_3/2_ peak shifted by 0.38 eV to a lower binding energy (707.14 eV). This pronounced shift in the chemical environment of the Fe sites confirms a strong chemical interaction between the pyrite surface and the oleate species, consistent with the formation of iron–oleate coordination complexes on the mineral surface [20].

### 3.7. Bench-Scale Tests

The metallurgical performance of the initial anionic reverse flotation stage (dolomite rejection) is presented in Table 3. Under the slightly acidic regime (pH 5.0), the MgO content was reduced from 2.75% in the feed to 1.14% in the phosphate concentrate, concurrently enriching the P_2_O_5_ grade from 23.38% to 26.23%. This validates the efficacy of NaOL in removing dolomite. Regarding sulfide mineral deportment, Pb and Zn contents in the initial feed were 42.36 ppm and 18.1 ppm, respectively. Following this stage, Pb and Zn were enriched in the froth product (tailings and middlings), with concentrations of 48.6 ppm Pb and 37.2 ppm Zn in the tailings, respectively. This actual ore behavior corroborates the single and mixed mineral tests confirming that a substantial portion of the sulfide minerals co-float with dolomite in the NaOL system.

The intermediate concentrate subsequently served as the feed for the cationic desilication stage (Table 4). The feed assay for this stage (41.2 ppm Pb, 13.78 ppm Zn) shows excellent agreement with the preceding stage’s sink product, ensuring rigorous mass balance consistency. Utilizing DTAB, the SiO_2_ content was substantially decreased from 15.16% to 10.33%, yielding a final concentrate with 31.47% P_2_O_5_. More importantly, the siliceous froth tailings were enriched to 44.9 ppm Pb and 16.1 ppm Zn. The Pb and Zn contents in the final phosphate concentrate were further reduced to 32.8 ppm Pb and 8.5 ppm Zn. The results show that the double-reverse flotation flowsheet possesses a “double-rejection” mechanism for sulfide minerals simultaneously with de-magnesium and desilication.

To further understand the deportment behavior, a size-by-assay analysis for Pb and Zn was conducted on the intermediate concentrate (Table 5). The data show an enrichment of both Pb and Zn in the fine particle fraction. Specifically, the −38 μm fraction, which accounts for 38.21% of the mass yield, contained the highest concentrations of Pb (42.8 ppm) and Zn (33.6 ppm). Conversely, the coarse fractions (−150 + 74 μm) contained lower concentrations. The results show that lead and zinc are preferentially enriched in the finer fractions.

**Figure 16:**
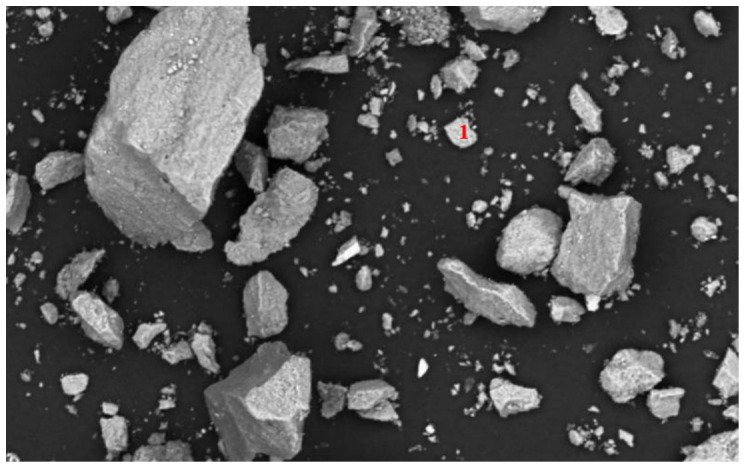
SEM image of the phosphate concentrate. The numbered points indicate specific mineral phases identified via EDS point analysis: 1 fluorapatite.

**Figure 17:**
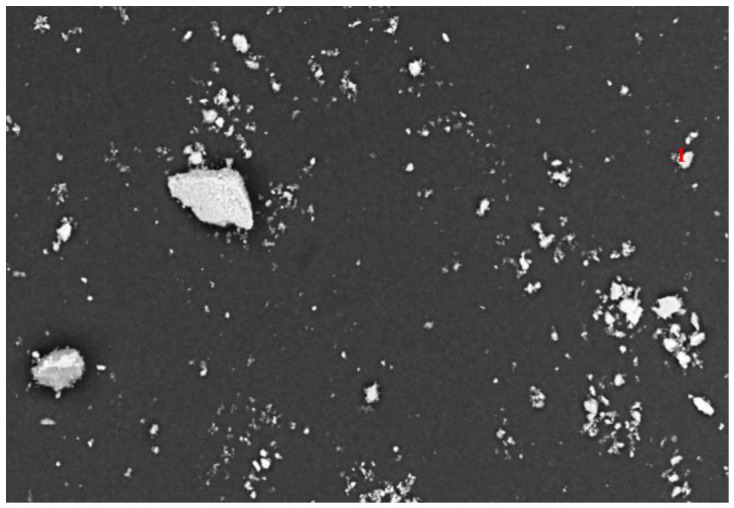
SEM image of the de-magnesium tailings. The numbered points indicate specific mineral phases identified via EDS point analysis: 1 dolomite.

**Figure 18:**
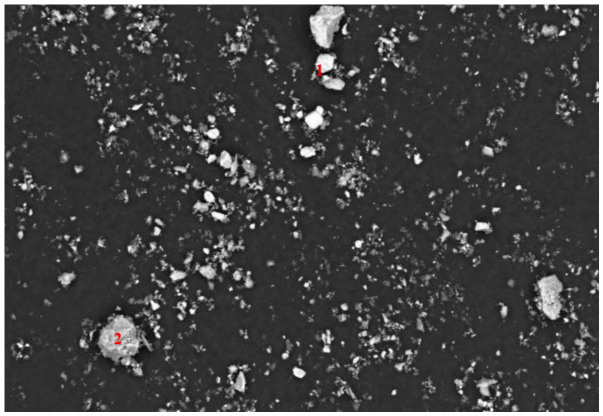
SEM image of the desilication tailings. The numbered points indicate specific mineral phases identified via EDS point analysis: 1 fluorapatite, 2 quartz.

**Figure 19:**
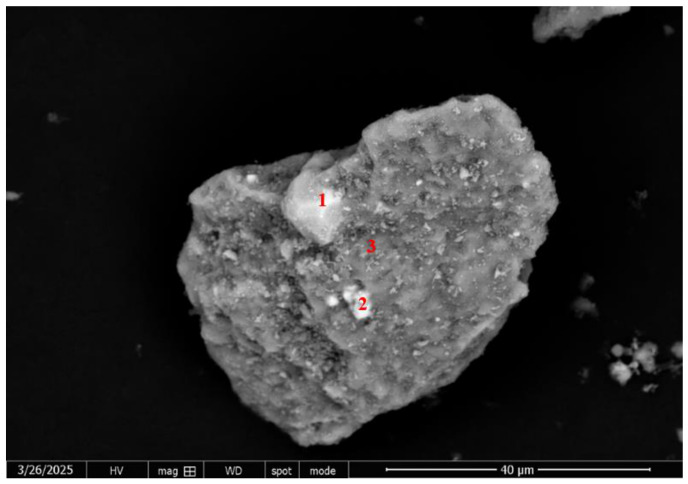
SEM image of the phosphate concentrate. The numbered points indicate specific mineral phases identified via EDS point analysis: 1 pyrite, 2 pyrite, and 3 fluorapatite.

To elucidate the fundamental limitations of this physical separation, the mineralogical associations of sulfide minerals in the flotation products were investigated via SEM-EDS (Figure 16, Figure 17, Figure 18 and Figure 19). Figure 16, Figure 17 and Figure 18 and Table 6, Table 7 and Table 8 show that a fraction of Pb and Zn is intricately interlocked with the major minerals including fluorapatite, dolomite and quartz, while Figure 19 and Table 9 specifically confirm the presence of pyrite. This interlocking phenomenon presents the ultimate sulfur distribution across the flowsheet. Carbon–sulfur analysis indicated that the sulfur grade was 0.715% in the carbonate tailings, 1.13% in the siliceous tailings, and 0.95% in the final concentrate. However, the presence of residual sulfur (0.95%) in the final concentrate delineates a critical boundary condition for this reagent regime. As corroborated by SEM-EDS analysis (Figure 16, Figure 17 and Figure 18), this residual impurity is fundamentally attributed to complex mineralogical interlocking rather than reagent inefficiency. This suggests that while the proposed double-rejection flowsheet is highly effective for adequately liberated particles, future optimization efforts should incorporate finer grinding or targeted pre-treatment strategies to further liberate these sulfide inclusions.

## 4. Conclusions

In this study, the characteristics and separation mechanisms of associated sulfide minerals during the acidic double-reverse flotation of phosphate ore were systematically investigated. The main conclusions are drawn as follows:

(1) Micro-flotation and surface analyses showed that NaOL functions as a strong collector for galena, sphalerite, and pyrite within the acidic window (pH 4.0–5.5). The oleate anions chemisorb onto the metallic active sites of the sulfides, as evidenced by significant binding energy shifts in XPS spectra (e.g., Pb 4f, Zn 2p, Fe 2p) and the formation of metal-carboxylate complexes. Conversely, DTAB interacts with the sulfides primarily through electrostatic attraction (physisorption).
(2) Artificial mixed mineral experiments showed that the strong chemisorption of NaOL induces a non-selective bulk flotation response, characterized by a high mass yield of 93.23%, where sulfides co-float with dolomite. In contrast, the electrostatically driven DTAB system exhibits superior selectivity with a precise mass yield of 54.91%. This confirms that DTAB effectively collects residual sulfides and siliceous gangue.
(3) Bench-scale tests on actual phosphate ore confirmed the practical efficacy of the proposed flowsheet. The identified “double-rejection” mechanism sequentially rejects sulfide minerals into the froth products of both the de-magnesium and desilication stages. Consequently, the P_2_O_5_ grade was significantly upgraded from 23.38% to 31.47%. However, size-by-assay analysis showed that Pb and Zn are preferentially enriched in the fine fraction (−38 μm), indicating that the extent of sulfide removal is inherently influenced by the degree of mineral liberation.

## Figures and Tables

**Table 1:** SEM-EDS elemental composition (wt.%) of the run-of-mine ore, corresponding to the point analyses in Figure 3.

Number	Element	Content (%)
1	S	11.38
	Fe	30.31
	Zn	14.81
	Pb	43.51
2	Fe	19.69
	S	2.94
	Pb	64.74
	Zn	12.62
3	Fe	47.43
	S	3.50
	Zn	30.15
	Pb	18.92

**Table 2:** Mass yield distribution of artificially mixed minerals using different collectors.

Mixed Mineral System	Collector	Dosage (mol/L)	pH	Yield of Froth Product (%)	Yield of Sink Product (%)
Mixture I (with Dolomite)	NaOL	1 × 10^−3^	5.0	93.23	6.77
Mixture II (with Quartz)	DTAB	1 × 10^−4^	5.0	54.91	45.09

**Table 3:** Metallurgical performance of the anionic reverse flotation stage for dolomite rejection and the partitioning of Pb and Zn.

Products	Yield	Assay						Lead Content	Zinc Content
	(%)	MgO	Fe_2_O_3_	Al_2_O_3_	SiO_2_	CaO	P_2_O_5_	(ppm)	(ppm)
Concentrate	79.9	1.14	2.43	4.29	17.49	37.73	26.23	40.7	13.3
Tailing	13.2	7.77	2.14	3.93	14.37	31.02	11.66	48.6	37.2
Middling	6.9	11.81	2.08	2.68	14.47	31.86	12.67	49.7	37.2
Feed	100	2.75	2.37	4.13	16.87	36.44	23.38	42.36	18.1

**Table 4:** Metallurgical performance of the cationic reverse flotation stage for desilication and the partitioning of Pb and Zn.

Products	Yield	Assay						Lead Content	Zinc Content
	(%)	MgO	Fe_2_O_3_	Al_2_O_3_	SiO_2_	CaO	P_2_O_5_	(ppm)	(ppm)
Concentrate	69.5	0.96	1.88	2.17	10.33	45.02	31.47	32.8	8.5
Tailing	30.5	7.40	2.38	4.17	26.14	29.40	19.85	44.9	16.1
Feed	100.0	2.93	2.03	2.78	15.16	40.25	27.92	41.2	13.78

**Table 5:** Lead and Zinc Content in Different Particle Size Fractions.

Size	Yield (%)	Lead Content (ppm)	Zinc Content (ppm)
−150 + 74 μm	18.65	28	21.3
−74 + 50 μm	26.91	29.2	22.9
−50 + 38 μm	14.26	29	26.2
−38 μm	38.21	42.8	33.6

**Table 6:** SEM-EDS elemental composition (wt.%) of the fluorapatite, corresponding to the point analyses in Figure 16.

Number	Element	Content (%)
1	O	59.182
	F	4.565
	P	11.277
	Ca	24.708
	Zn	0.221
	Pb	0.047

**Table 7:** SEM-EDS elemental composition (wt.%) of the dolomite, corresponding to the point analyses in Figure 17.

Number	Element	Content (%)
1	C	27.427
	O	55.353
	Mg	7.549
	Ca	9.610
	Zn	0.055
	Pb	0.017

**Table 8:** SEM-EDS elemental composition (wt.%) of the fluorapatite and quartz, corresponding to the point analyses in Figure 18.

Number	Element	Content (%)
1	O	65.102
	F	6.276
	P	9.223
	Ca	19.141
	Zn	0.172
	Pb	0.087
2	O	44.061
	Mg	1.137
	Al	24.260
	Si	28.706
	Fe	1.650
	Zn	0.141
	Pb	0.044

**Table 9:** SEM-EDS elemental composition (wt.%) of the pyrite and fluorapatite, corresponding to the point analyses in Figure 19.

Number	Element	Content (%)
1	S	46.83
	Fe	43.72
	Zn	0.04
2	Fe	40.76
	S	38.87
3	O	27.37
	Ca	47.19
	Fe	0.96
	F	1.96
	P	17.19
	C	5.23
	Zn	0.06
	Pb	0.04

## Data Availability

The original contributions presented in this study are included in the article. Further inquiries can be directed to the corresponding authors.

## References

[1] Farid Z., Abdennouri M., Barka N., Sadiq M. (2025). Current and sustainable approaches in phosphate ore flotation: A review of eco-friendly reagents and their applications. J. Ind. Eng. Chem..

[2] Yu L., Yu P., Bai S. (2024). A Critical Review on the Flotation Reagents for Phosphate Ore Beneficiation. Minerals.

[3] Derhy M., Taha Y., Hakkou R., Benzaazoua M. (2020). Review of the Main Factors Affecting the Flotation of Phosphate Ores. Minerals.

[4] Amirech A., Bouhenguel M., Kouachi S. (2018). Two-stage reverse flotation process for removal of carbonates and silicates from phosphate ore using anionic and cationic collectors. Arab. J. Geosci..

[5] Huang X., Zhang Q. (2024). Depression mechanism of acid for flotation separation of fluorapatite and dolomite using ToF-SIMS and XPS. J. Mol. Liq..

[6] Zou H., Cao Q., Liu D., Yu X., Lai H. (2019). Surface Features of Fluorapatite and Dolomite in the Reverse Flotation Process Using Sulfuric Acid as a Depressor. Minerals.

[7] Xu W., Mei G., Tian Y., Shi B., Guo C., Pan W. (2024). Reverse cationic flotation of low-grade phosphate ore using quaternary ammonium salt as a collector and its adsorption mechanism. Green Smart Min. Eng..

[8] Mohammadkhani M., Noaparast M., Shafaei S.Z., Amini A., Amini E., Abdollahi H. (2011). Double reverse flotation of a very low grade sedimentary phosphate rock, rich in carbonate and silicate. Int. J. Miner. Process..

[9] Ahmadi N., Felhi M., El Bahri D., Molina-Piernas E., Chebbi N., Tlili A. (2025). Beneficiation of low-grade carbonated phosphate ore by reverse flotation technics using anionic and cationic collectors, Sra Ouertane region, Northwest Tunisia. Euro-Mediterr. J. Environ. Integr..

[10] Suciu N.A., De Vivo R., Rizzati N., Capri E. (2022). Cd content in phosphate fertilizer: Which potential risk for the environment and human health?. Curr. Opin. Environ. Sci. Health.

[11] Ahmad N., Usman M., Ahmad H.R., Sabir M., Farooqi Z.U.R., Shehzad M.T. (2023). Environmental implications of phosphate-based fertilizer industrial waste and its management practices. Environ. Monit. Assess..

[12] Zhao Y., Li X., Yu J., Li C., Ruan Y., Abbas M.A., Chi R. (2025). Migration and transformation behaviors of phosphorus and associated elements in wet-process phosphoric acid: Acidolysis process and mechanism study. J. Environ. Chem. Eng..

[13] Huo X., Guo L., Liu R., Tao C., Xi B. (2023). Role of Additives: Modified Hemihydrate Phosphogypsum Morphology and Enhanced Filtration Performance of Wet-Process Phosphoric Acid. ACS Omega.

[14] Shariati S., Ramadi A., Salsani A. (2015). Beneficiation of Low-Grade Phosphate Deposits by a Combination of Calcination and Shaking Tables: Southwest Iran. Minerals.

[15] Pashkevich D., Li R., Kökkılıç O., Waters K.E. (2023). Investigations of Monomineralic Flotation of Galena, Sphalerite, and Pyrite at Different Temperatures. Minerals.

[16] Tang X., Long Q., Chen J., Chen Y. (2024). An in-situ study of the interaction mechanism between xanthate and unactivated/Cu-activated sphalerite surfaces at solid–liquid interfaces. Appl. Surf. Sci..

[17] Miao Y., Ye G., Zhang G. (2024). Effect of dissolved-oxygen on the flotation behavior of pyrite at high altitude area. Int. J. Miner. Metall. Mater..

[18] Cheng W., Deng Z., Tong X., Lu T. (2020). Hydrophobic Agglomeration of Fine Pyrite Particles Induced by Flotation Reagents. Minerals.

[19] Paredes A., Acuña S.M., Gutiérrez L., Toledo P.G. (2019). Zeta Potential of Pyrite Particles in Concentrated Solutions of Monovalent Seawater Electrolytes and Amyl Xanthate. Minerals.

[20] Dong Y., Liu Z., Lin H. (2023). Hydrophobic modification of pyrite with a composite of sodium oleate and SiO_2_ nanoparticles to inhibit its oxidation for controlling acid mine drainage. J. Environ. Chem. Eng..

[21] Jin J., Lai H., Xiao Y., Lu X., Shen P., Cai J., Wei X., Liu D. (2026). Adsorption mechanism of polyepoxysuccinic acid on the sphalerite surface and its effect on flotation separation. J. Taiwan Inst. Chem. Eng..

[22] Liu W., Huang W., Rao F., Zhu Z., Zheng Y., Wen S. (2022). Utilization of DTAB as a collector for the reverse flotation separation of quartz from fluorapatite. Int. J. Miner. Metall. Mater..

[23] Salako O.I., Gonzalez J.J., Cummins A.G., Zambrano J.L., Lewis B., Lentz R., Lin Z., Aghadiuno P.O., Stinson W.H., Esposito D.V. (2026). Zeta potential measurements of SiO_2_ and TiO_2_ particles in anionic and cationic surfactant solutions. Surf. Interfaces.

[24] Khan S.A., Khan S.B., Khan L.U., Farooq A., Akhtar K., Asiri A.M., Sharma S.K. (2018). Fourier Transform Infrared Spectroscopy: Fundamentals and Application in Functional Groups and Nanomaterials Characterization. Handbook of Materials Characterization.

[25] Borda M.J., Strongin D.R., Schoonen M.A. (2004). A vibrational spectroscopic study of the oxidation of pyrite by molecular oxygen. Geochim. Cosmochim. Acta.

[26] Jin J., Gao H., Ren Z., Chen Z. (2016). The Flotation of Kyanite and Sillimanite with Sodium Oleate as the Collector. Minerals.

[27] Creutzburg M., Konuk M., Tober S., Chung S., Arndt B., Noei H., Meißner R.H., Stierle A. (2022). Adsorption of Oleic Acid on Magnetite Facets. Commun. Chem..

[28] Wagner M., Pigliapochi R., Di Tullio V., Catalano J., Zumbulyadis N., Centeno S.A., Wang X., Chen K., Hung I., Gan Z. (2023). Multi-technique Structural Analysis of Zinc Carboxylates (Soaps). Dalton Trans..

[29] Liu C., Feng Q., Zhang G., Ma W., Meng Q., Chen Y. (2016). Effects of Lead Ions on the Flotation of Hemimorphite Using Sodium Oleate. Miner. Eng..

[30] Lv S., Liang Y., Zhang X., Tan X., Huang Z., Guan X., Liu C., Tu Z. (2024). An Electrochemical Study of the Effect of Sulfate on the Surface Oxidation of Pyrite. Materials.

[31] Liu W., Tong K., Ding R., Liu W., Zhao P., Sun W., Zhao Q., Zhao S. (2023). Synthesis of a Novel Hydroxyl Quaternary Ammonium Collector and Its Selective Flotation Separation of Quartz from Hematite. Miner. Eng..

[32] Liu X., Du Z., Sun C., Zhang N. (2025). A Review on the Electrochemical Analysis of Sulfide Minerals—Pyrite, Chalcopyrite, and Galena. Green Smart Min. Eng..

[33] Chai W., Zhang H., Zhang H., Cao Y., Chen Y. (2025). Selective Adsorption Behaviors and Mechanisms of Benzyl Quaternary Ammonium Salt on Quartz and Gypsum: A Combined Experimental and Theoretical Study. Colloids Surf. A Physicochem. Eng. Asp..

[34] Grey L.H., Nie H.-Y., Biesinger M.C. (2024). Defining the Nature of Adventitious Carbon and Improving Its Merit as a Charge Correction Reference for XPS. Appl. Surf. Sci..

[35] Bai S., Li J., Bi Y., Yuan J., Wen S., Ding Z. (2023). Adsorption of Sodium Oleate at the Microfine Hematite/Aqueous Solution Interface and Its Consequences for Flotation. Int. J. Min. Sci. Technol..

[36] Ding Z., Li J., Yuan J., Yu A., Wen S., Bai S. (2023). Insights into the Influence of Calcium Ions on the Adsorption Behavior of Sodium Oleate and Its Response to Flotation of Quartz: FT-IR, XPS and AMF Studies. Miner. Eng..

[37] Fu X., Gao Y., Peng C., Han H., Sun W., Yue T. (2024). Adsorption Mechanism of Sodium Oleate at Hematite/Quartz–Water Interfaces: A Quantitative Molecular Insight. Miner. Eng..

[38] Feng Y., Chen Y., Chen J. (2024). The Mechanism of Surface Activation in Sphalerite by Metal Ions with d10 Electronic Configurations: Experimental and DFT Study. Appl. Surf. Sci..

[39] Liu R., Zhang H., Dong W., Sun W. (2026). Efficient Separation of Sphalerite and Galena: Inhibition of Sphalerite by Disodium Hydrogen Phosphate Driven by Zinc Site Chelation. Miner. Eng..

[40] Nesbitt H.W., Scaini M., Höchst H., Bancroft G.M., Schaufuss A.G., Szargan R. (2000). Synchrotron XPS Evidence for Fe^2+^-S and Fe^3+^-S Surface Species on Pyrite Fracture-Surfaces, and Their 3D Electronic States. Am. Mineral..

